# Miniaturized NIR Spectrometer Based on Novel MOEMS Scanning Tilted Grating

**DOI:** 10.3390/mi9100478

**Published:** 2018-09-20

**Authors:** Jian Huang, Quan Wen, Qiuyu Nie, Fei Chang, Ying Zhou, Zhiyu Wen

**Affiliations:** 1 Key Laboratory of Fundamental Science of Micro/Nano-Device and System Technology, Chongqing University, Chongqing 400044, China; qiu30@163.com (Q.N.); changfei0602@gmail.com (F.C.); yzhou@cqu.edu.cn (Y.Z.); wzy@cqu.edu.cn (Z.W.); 2 College of Information Engineering, Qujing Normal University, Qujing 655000, China; 3 Microsystem Research Center, College of Optoelectronic Engineering, Chongqing University, Chongqing 400044, China; 4 Fraunhofer Institute for Electronic Nano Systems (ENAS), 09131 Chemnitz, Germany; 5 College of Electronic and Information Engineering, Southwest University, Chongqing 400715, China

**Keywords:** micro-NIR spectrometer, scanning grating mirror, deflection position detector

## Abstract

This paper presents a dispersive near-infrared spectrometer with features of miniaturization, portability and low cost. The application of a resonantly-driven scanning grating mirror (SGM) as a dispersive element in a crossed Czerny–Turner configuration enables the design of a miniaturized spectrometer that can detect the full spectra using only one single InGaAs diode. In addition, a high accuracy recalculation is realized, which can convert time-dependent measurements to spectrum information by utilizing the deflection position detector integrated on SGM and its associated closed-loop control circuit. Finally, the spectrometer prototype is subjected to a series of tests to characterize the instrument’s performance fully. The results of the experiment show that the spectrometer works in a spectral range of 800 nm–1800 nm with a resolution of less than 10 nm, a size of 9 × 7 × 7 cm^3^, a wavelength stability better than ±1 nm and a measuring time of less than 1 ms. Furthermore, the power consumption of the instrument is 3 W at 5 V DC, and the signal-to-noise ratio is 3267 at full scale. Therefore, this spectrometer could be a potential alternative to classical spectrometers in process control applications or could be used as a portable or airborne spectroscopic sensor.

## 1. Introduction

Because of their ability to detect the composition and content of substances non-destructively, near-infrared spectrometers have a wide range of applications [1]. However, due to their disadvantages in terms of volume, power consumption, portability and price, classical spectrometers have limited application in some fields, like remote sensing, in-field spectroscopy, astronomy, analytical chemistry and process control. Therefore, the miniaturization of the NIR spectral instruments has become a development trend and has an urgent application requirement.

MEMS and MOEMS technology have experienced rapid progress in recent decades. In the field of NIR spectroscopy, MEMS- and MOEMS-based NIR spectrometers have made important contributions to the process of instrument miniaturization. The incorporation of MEMS- or MOEMS-based devices into NIR spectrometers has become one of the research hotspots in the field of spectroscopic instruments, and such devices are characterized by cost effectiveness, portability, low power consumption, high speed and small size [2,3].

Since linear array detectors are expensive in the near-infrared band, in order to reduce the cost and size of the spectrometer, MOEMS-based NIR spectral instruments are usually designed with integrated movable scanning components so that a single-tube detector can be used to obtain the entire spectrum. This type of spectrometer can be classified into two categories based on spectroscopic principles: (1) nondispersive methods and (2) dispersive methods. The typical representatives of the former are the Fourier transform spectrometer (FTS) [4], the Fabry–Perot interferometer (FPI) [5,6], the grating light modulator spectrometer [7] and the quantum dot spectrometer [8]. Sandner proposed a Fourier transform spectrometer that utilizes an out-of-plane MEMS mirror in 2007 [9]. An FTS has several basic advantages, including a high signal-to-noise ratio (SNR) and linearity and a broad wavelength range. It also has disadvantages, such as light fluctuation, which creates noise at all wavelengths, and the need for significant amount of data analysis. A major hurdle, especially for MEMS, is that the FTS requires significant movement of reflecting surfaces, which miniaturized devices cannot easily achieve. Malinen proposed a piezo-actuated NIR FPI in 2010 [10]. This kind of instrument needs to be mass manufactured in high volumes to drive down the cost of individual FPI chips.

Kraft proposed a single-detector micro-electro-mechanical scanning grating spectrometer in 2006 [11], which combines the features of a spectrometer monochromator with the advantages of optical MEMS components. Spectral scanning can be accomplished by the rotation of an oscillating reflection grating, which belongs to the grating dispersion type. Because scanning grating is not integrated with any position detecting device, a laser projection system is used to detect the deflection angle of the dispersive element, which would enlarge and complicate the entire system. Furthermore, an additional deflection position detection device results in an increase in instrument volume, which cannot meet the special application requirements of the micro-NIR spectrometer based on the MOEMS scanning grating mirror. In addition, the signal-to-noise ratio is relatively low because blazed grating is not introduced.

The spectrometer presented in this paper was designed and implemented based on an MOEMS scanning grating micro-mirror, integrated with a deflection position detector. The angle signal is generated synchronously with the movement of the mirror, which serves as a trigger signal of the spectrum A/D acquisition and a reference signal of the mirror closed loop control. Therefore, the scanning range can be dynamically adjusted according to the respective requirements. In addition, the spectral changes can be monitored with a high time resolution (less than 1 ms), and the driving voltage is lower than 1 V.

The optical design and spectral reconstruction of the spectrometer are introduced in Section 2. After this, the system characterization is described in Section 3. Finally, a brief conclusion is given in Section 4.

## 2. System Design

The spectrometer based on MOEMS technology consists of four main parts, including the fabrication of MOEMS core components, optical design, circuit design and spectral reconstruction.

This paper focuses mainly on optical design, system integration and spectral reconstruction.

### 2.1. MEMS Component

The scanning dispersive element is the key component in the spectrometer system. It is vital to have a small and robust movable diffraction grating device.

**Figure 1:**
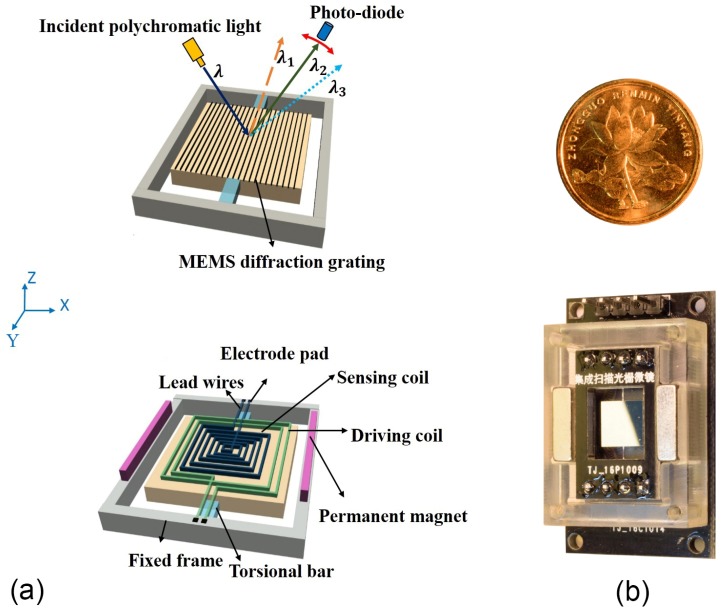
Schematic of the electromagnetic MEMS scanning mirror. (**a**) The integrated blazed grating (front) and driving and sensing coils (back); (**b**) scanning grating micro-mirror component.

Two kinds of devices were fabricated by Chongqing University Microsystem Research Center based on different materials, including single crystalline silicon [12,13,14] and FR4 [15]. To take thermal sensitivity into account, the Si MEMS electromagnetic scanning grating mirror (SGM) was selected as the scanning tilted grating. The movable mirror plate is suspended inside a frame by two rectangular torsion bars. One side of the movable plate is integrated with the blazed grating, and the other is integrated with the driving and sensing coils, as shown in Figure 1a. The specific parameters of the SGM are shown in Table 1. The corresponding signal processing circuits were also prepared [16]. When the component was driven at a voltage of 650 mV and a frequency slightly above the resonance frequency, e.g., at 620 Hz, its mechanical deflection angle was shown to reach ±4°. As a result of the combination of the optical layout and mirror blazed grating (period of 4 μm), the scanning wavelength range can cover 800–1800 nm.

### 2.2. Optical Design

Due to its compact size and ability to suppress stray light effectively, the Czerny–Turner system has become the classical structure of commercial spectrometers. This paper presents a modified version of the Czerny–Turner system. First, there is light crossing in this layout, which further reduces the instrument’s volume. In addition, the focal lengths of the focusing mirror and the collimation mirror are not equal. This eliminates coma aberration at the specified wavelength. It is an asymmetrical crossed Czerny–Turner system with a 48° total deflection angle between the incident and refracted beam.

**Figure 2:**
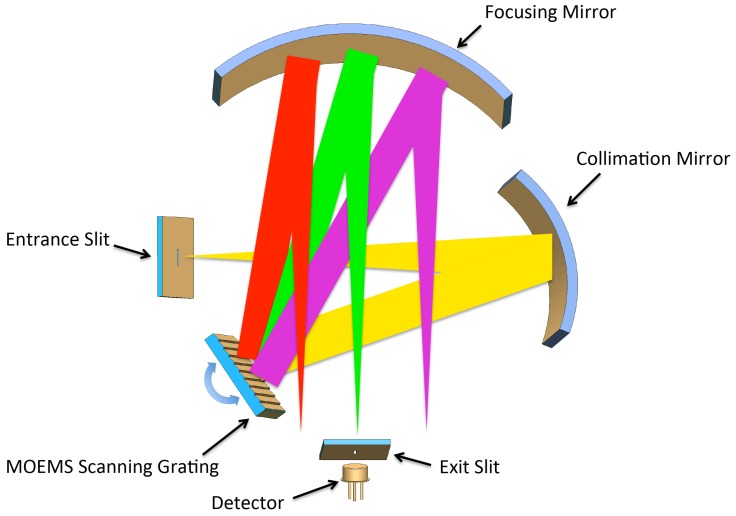
Principal drawing of the spectrometer showing the asymmetrical crossed Czerny–Turner setup.

The 3D optical layout is shown in Figure 2. The optics system includes an entrance slit, collimating mirror, scanning grating mirror (SGM), focusing mirror, exit slit and detector. In this optics system, the light radiation to be analyzed passes through a vertical 50-μm entrance slit aperture, collimated by a spherical mirror (focal length of 50 mm). It is diffracted by the scanning grating and focused again by a second spherical mirror (focal length of 75 mm) to the exit slit, and then, it reaches the detector (Hamamatsu G12181-05). Between the exit slit and focusing mirror, the spectrometer is equipped with an 800-nm long-pass filter as the second-order filter. The specific design parameters of the spectrometer are shown in Table 2.

In addition to the portability and compactness of the spectrometer, another important feature is the scanning speed. When the scanning grating micromirror oscillates at a resonant frequency of 620 Hz, the time taken for a single spectral scan is only 0.83 ms. The fast scanning capability can be applied to either monitor the spectral signal changes at a high speed or to reduce the effection of random noise by co-adding multiple times in a short time period.

### 2.3. The Spectra Reconstruction

**Figure 3:**
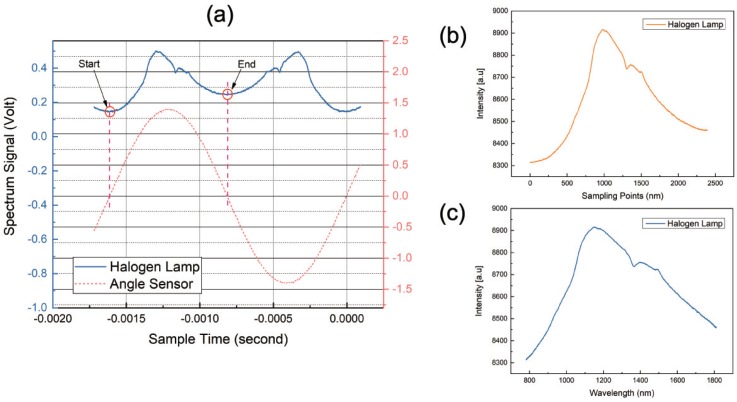
(**a**) Two full scans within one mirror oscillation; (**b**) time-dependent spectrum of a halogen lamp and (**c**) wavelength-dependent spectrum of a halogen lamp using reconstruction.

There are two full scans within one mirror oscillation, forwards and backwards, as shown in Figure 3a. Therefore, it is enough to acquire one spectrum of the forward scan. The return point of the mirror is the correct starting point for sampling a new spectrum.

Spectrum acquisition with one single detector is always a time-dependent measurement. When the spectrometer circuit is working, A/D sampling will be performed on the single-tube detector to obtain the relationship between light intensity and time; it is I(t), as shown in Figure 3b.

During the movement of the scanning grating mirror, the change in the deflection angle with time is nonlinear. Therefore, it is necessary to unfold the spectrum in the wavelength domain by means of the deflection position signal and the basic grating equation.

The maximum deflection angle of the mirror can be obtained from the deflection position detector. Together with the known sinusoidal movement of the mirror over time, ϕ(t) can be obtained with:

(1) $$\phi \left(t\right)=\phi_{max}\cdot \cos\left(2\cdot \pi \cdot f_{mirror}\cdot t\right).$$

The deflection position detector shows the mark of the spectra. There is a linear relationship between the deflection angle and the wavelength. By using the combined grating equation, the wavelength sliding over the detector at a certain timestamp can be determined. It is λ(t) and is described as:

(2) λ(t)=g·(sinβ(t)−sinα(t))

where:

(3) $$\alpha \left(t\right)=\alpha_{middle}+\phi \left(t\right)$$

(4) $$\beta \left(t\right)=\beta_{middle}-\phi \left(t\right)$$

(5) $$\beta_{middle}=arcsin\left(\frac{\lambda_{middle}}{g}+\sin\alpha_{middle}\right)$$

where $\alpha_{middle}$, $\beta_{middle}$, $\lambda_{middle}$ and *g* represent the angle of incidence, the angle of diffraction, the wavelength when the grating mirror in its non-deflected position and the grating constant.

The final spectrum I(λ) can be calculated by I(t) and λ(t), as shown in Figure 3c.

## 3. System Characterization

The experimental program followed the classical arrangement (light source-sample-fiber optics-spectrometer), with a 400 μm (NA=0.22) low-OH silica fiber. All tests were performed at room temperature, i.e., 22 ± 2 °C, unless otherwise specified.

### 3.1. Wavelength Range

**Figure 4:**
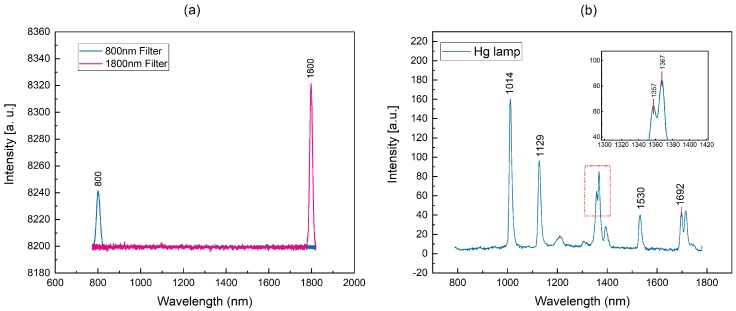
(**a**) Wavelength range; (**b**) spectral resolution.

While the mechanical deflection angle reaches ±4°, the spectral scanning range can cover 800–1800 nm, as shown in Figure 4a. Since the photosensitivity of InGaAs detector varies at each wavelength, the light intensities of the 800-nm filter and the 1800-nm filter are different.

As the prominent bands are 1446 nm for water and 1650 nm–1800 nm for carbon compounds, the 800-nm–1800-nm range is appropriate. Even though some interesting bands can be detected between 1900 nm and 2500 nm, the additional effort required for detector cooling increases system complexity and is not suitable for portable applications.

### 3.2. Spectral Resolution

To evaluate the effective spectral resolution of the spectrometer prototype, a special test setup was established in the laboratory. Light from a standard high pressure mercury lamp was coupled to the fiber of the spectrometer. The result of the measurement is depicted in Figure 4b. It shows the whole spectral range from 800 nm–1800 nm and a magnified section of two adjacent peaks spaced 10 nm apart [17]. Obviously, the spectrometer prototype can distinguish two peaks at 1357 nm and 1367 nm. According to Rayleigh’s criterion, the resolution of the spectrometer is better than 10 nm.

In addition, the theoretical spectral resolution can be calculated by Equation (6).

(6) $$\delta \lambda =\frac{W_{slit}\cdot g\cdot cos\alpha }{m\cdot L_{c}}$$

where $W_{slit}$ is the width of the entrance slit; *m* is the spectral order; $L_{c}$ is the focal length of the collimating lens; *g* is the grating constant; and α is the incidence of grating. The theoretical resolution and the measured resolution are shown in Table 3.

Because of assembly errors, part machining errors and system aberrations, the measured resolution is larger than the theoretical value. In practical applications of NIR spectroscopy, the requirements for spectral resolutions in 800–1800 nm are intermediate due to the broad structure of the over-tone and combination bands. Therefore, for most applications of an NIR spectrometer as a pocket-sized or handheld spectra analyzer, this resolution (10 nm) is at a good enough level.

### 3.3. Wavelength Stability

From the perspective of chemometrics, 1 nm is the minimum requirement for long-term stability.

**Figure 5:**
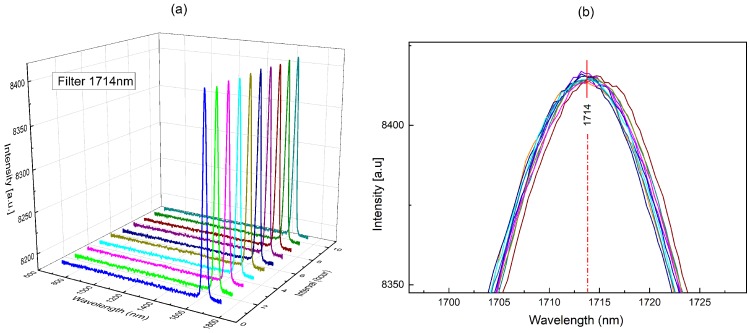
(**a**) Three-dimensional schematic of stability testing; (**b**) 10 measurements around the central wavelength of a 1714-nm filter.

In order to obtain accurate measurement data in terms of wavelength stability, a 1714-nm interference band-pass filter illuminated by a tungsten halogen lamp was tested 10 times every 15 min, as shown in Figure 5a. By analyzing the degree of deviation of the 10 measurements from the center wavelength (1714 nm), it was concluded that the wavelength stability was better than ±1 nm, meeting the requirement for long-term stability, as shown in Figure 5b.

### 3.4. Signal-to-Noise Ratio Characteristics

For the reliable evaluation of spectra, it is important to ensure a sufficient signal level and low noise. Independent of the spectral setup, the signal-to-noise ratio (SNR), typically given for the lowest signal level that can be detected, contributes to the performance description of a spectrometer.

The SNR improves with an increasing number of co-added scans [11]. For the spectrometer prototype described in this paper, when sampling 50 times for averaging, the signal-to-noise ratio is 1700, and a single scan acquired within 0.8 ms is noisier, about 200. When the light source is strong enough to make the spectrometer work at full scale and the co-added scans exceed 100 times, the signal-to-noise ratio can reach 3267, as shown in Table 4. As a handheld instrument, this is satisfactory for most NIR applications.

**Figure 6:**
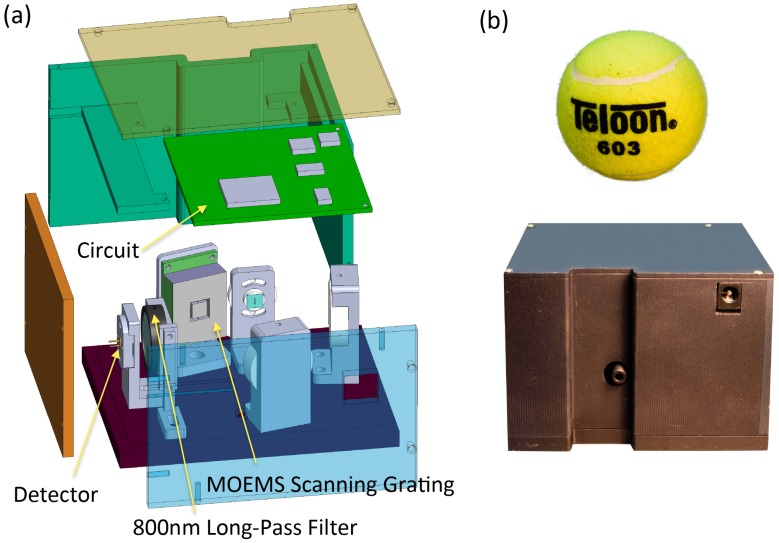
(**a**) Expanded assembly drawing of the spectrometer; (**b**) a single-detector NIR microspectrometer encased in an aluminum housing, which is comparable in size to a tennis ball.

Packaged in an aluminum shell with a three-dimensional size of 90 × 70 × 70 mm^3^, the resulting spectrometer prototype has a weight of approximately 0.65 kg and a power consumption of less than 3 W at 5 V DC. It is comparable in size to a tennis ball (Figure 6b).

The power consumption and weight of the MEMS spectrometer in comparison with the SGS 1900 [11] and irSys E2.1 [18,19] are listed in Table 5.

## 4. Conclusions

In summary, a prototype of a miniaturized NIR spectrometer based on novel MOEMS scanning grating integrated with a deflection position detector was presented in this paper. As a scanning grating Czerny–Turner structure spectrometer, it has the characteristics of miniaturization, low power consumption and low cost when introducing the MOEMS device as the core device. A series of test results showed that the spectrometer’s scanning range can cover 800–1800 nm with a single scan time of less than 1 ms, a spectral resolution of better than 10 nm and a wavelength stability of better than ±1 nm. In addition, the signal-to-noise ratio was shown to be sufficient to satisfy most NIR analyses. In the future, the performance of the instrument could be further improved by employing a parabolic collimation mirror while controlling the magnification of the optical system so that the exit slit can be removed to improve the signal-to-noise ratio.

## Figures and Tables

**Table 1:** Scanning tilted grating parameters.

Parameter	Value
Resonance frequency (Hz)	620 Hz
Mechanical deflection angle (°)	±4 (driving voltage: 650 mV)
Mirror plate (mm^2^)	6
Quality factor (Q) (a.u.)	125
Grating groove density (lines/mm)	250
Blazed angle (°)	7.9

**Table 2:** Spectrometer parameters.

Parameter	Value
Object distance (mm)	50
Wavelength range (nm)	800–1800
Entrance slit (μm)	50
Exit slit (μm)	85
NA (a.u.)	0.22
Image distance (mm)	75
Center wavelength (nm)	1300
Angle of incidence (°)	14
Diffraction order (a.u.)	+1

**Table 3:** Comparison of the theoretical resolution with the measured resolution.

Parameter	Theoretical	Measured
Resolution (nm)	3.9	10

**Table 4:** Standard deviation, peak intensity and SNR of the spectrum of a tungsten halogen lamp source with 1, 50 and 100 averaging.

Parameter	1×	50×	100×
Baseline intensity SD (a.u.)	0.0049	0.0006	0.0003
Peak intensity (a.u.)	0.9913	0.9874	0.9802
SNR (a.u.)	200:1	1700:1	3267:1

**Table 5:** Important parameters of the microspectrometer prototype and the corresponding values for the Hiperscan SGS 1900 and irSys E2.1 for comparison.

Parameter	Microspectrometer	SGS 1900	irSys E2.1
Wavelength range (nm)	800–1800	1200–1900	910–2100
Spectral resolution (nm) *	10	10	11
Volume (cm^3^)	441	600	810.6
SNR (a.u.) ^†^	3267:1	1700:1	2500:1
Power consumption (W) ^‡^	3	5	5
Scan time (ms)	0.83	4	4
Power supply (V)	5	7.5	24

* 50-μm entrance slit; ^†^ measured at 1650 nm with 100 averaging; ^‡^ including complete readout electronics.
